# Precocious puberty and microbiota: The role of the sex hormone–gut microbiome axis

**DOI:** 10.3389/fendo.2022.1000919

**Published:** 2022-10-21

**Authors:** Valeria Calcaterra, Virginia Rossi, Giulia Massini, Corrado Regalbuto, Chiara Hruby, Simona Panelli, Claudio Bandi, Gianvincenzo Zuccotti

**Affiliations:** ^1^ Pediatric Department, “Vittore Buzzi” Children’s Hospital, Milan, Italy; ^2^ Department of Internal Medicine, University of Pavia, Pavia, Italy; ^3^ Pediatric unit , Fondazione Istituto di Ricovero e Cura a Carattere (IRCCS) Policlinico S. Matteo and University of Pavia, Pavia, Italy; ^4^ Pediatric Clinical Research Center “Invernizzi”, Department of Biomedical and Clinical Sciences “L. Sacco”, University of Milan, Milan, Italy

**Keywords:** precocious puberty (PP), gut, microbioma, sex hormone, axis

## Abstract

Puberty is a critical phase of life associated with physiological changes related to sexual maturation, and represents a complex process regulated by multiple endocrine and genetic controls. Puberty is driven by hormones, and it can impact the gut microbiome (GM). GM differences between sex emerge at puberty onset, confirming a relationship between microbiota and sex hormones. In this narrative review, we present an overview of precocious pubertal development and the changes in the GM in precocious puberty (PP) in order to consider the role of the sex hormone–gut microbiome axis from the perspective of pediatric endocrinology. Bidirectional interactions between the GM and sex hormones have been proposed in different studies. Although the evidence on the interaction between microbiota and sex hormones remains limited in pediatric patients, the evidence that GM alterations may occur in girls with central precocious puberty (CPP) represents an interesting finding for the prediction and prevention of PP. Deepening the understanding of the connection between the sex hormones and the role of microbiota changes can lead to the implementation of microbiota-targeted therapies in pubertal disorders by offering a pediatric endocrinology perspective.

## Introduction

Puberty is a critical phase of life associated with physiological changes related to sexual maturation and represents a complex process regulated by multiple endocrine and genetic controls (1). Gonadal sex hormones are secreted in accordance with the pulsatile secretion of the pituitary gonadotropin follicle-stimulating hormone and luteinizing hormone (FSH and LH), which is activated by the release of the hypothalamic gonadotropin releasing hormone (GnRH) (2). Rising levels of sex steroid hormones trigger changes in physical appearance during pubertal development.

Pubertal development can be at the appropriate time, precocious or delayed. Abnormal pubertal development can cause considerable distress to the patient and could also be a sign of an underlying pathology.

Puberty is driven by hormones, and it can impacts the gut microbiome (GM) (3). Gut microbial communities represent one source of human genetic and metabolic diversity; they influence nutrient acquisition, brain development, immunity, endocrinology and the nervous system (4–6). Few studies have described changes in the gut microbiome as a function of age. It has been observed that the development of the microbiome from infancy to childhood is dependent on multiple factors, and an association between sex hormones and microbiota has been proposed. GM differences between sex emerge at puberty onset, confirming a relationship between microbiota and sex hormones (3, 7). Some theories suggest that the GM regulates the levels of sex hormones via interactions among its metabolites, the immune system, chronic inflammation and some nerve–endocrine axes, such as the gut–brain axis. Additionally, bidirectional interactions between the microbiome and the hormonal system have also been proposed, and the mechanisms of these interactions are being explored; data are limited in pediatrics (8).

In this narrative review, we presented an overview on the precocious pubertal development and the changes in the GM in precocious puberty in order to consider the role of the sex hormone–gut microbiome axis from the perspective of pediatric endocrinology.

## Methods

A narrative review was presented (9); a non-systematic summation and analysis of the available literature on the topic of the changes in the GM in precocious puberty and the role of the sex hormone–gut microbiome axis was considered. Authors reviewed the relevant English literature on a specific topic, in the past 15 years, including original papers, metanalysis, clinical trials and reviews. Case reports and case series were excluded. A search in PubMed, Scopus, EMBASE, and Web of Science was adopted. The following search terms (alone and/or in combination) were adopted: precocious puberty, timing of puberty, gut microbiome, sex hormones, sex hormone–gut microbiome axis. The authors revised the abstracts of the available literature (n=83) and reviewed the full texts of potentially relevant articles (n=50) that were analyzed and critically discussed. The reference list of all articles was checked to consider relevant studies. The contributions were independently collected by V.R., G.M., C.R. and C.H., critically analyzed by V.C., S.P. and V.C., S.P., C.B., G.Z discussed the resulting draft before finalizing. The final version was approved by all the coauthors.

## Pubertal development

### Physiology of the puberty

Puberty is a crucial developmental milestone characterized by the maturation of gametogenesis (precursor cells undergo cell division and differentiation to form mature haploid gametes), the production of gonadal hormones and the development of secondary sexual characteristics and reproductive functions.

Normal puberty results from prolonged, mature activity of the hypothalamic–pituitary–gonadal (HPG) axis (2). The hypothalamus releases the GnRH in a pulsatile way into the pituitary portal venous system, where it stimulates LH and FSH secretion (pulsatile as well). LH primarily stimulates Leydig cells in the testes and theca cells in the ovary to secrete androgens. FSH primarily stimulates the ovarian follicle or seminiferous tubules to form estrogen, inhibin, and eggs or sperm. The interstitial, tubular and follicular compartments act together through paracrine processes to produce estrogen, and regulate sex steroid production and gamete development. Steroid hormones have endocrine negative feedback effects on GnRH and gonadotropin secretion. FSH secretion is suppressed by the negative feedback of inhibin, progesterone, and estradiol. In adult female subjects, critical estradiol concentration stimulates the LH surge that initiates ovulation.

At birth, due to the absence of placental steroids that suppress the HPG axis, there is an activation of such axis, which causes an increased production of steroidal hormones, defining the first step that will be continued in adolescence. This transient activation starts approximately one week after birth, and it stops after few months (approximately 6 months of age) (10–12).

The HPG axis is not completely dormant throughout childhood, especially in females, who show moderately higher FSH concentrations than males. Sometimes, it is also possible to see ovarian follicles using ultrasounds. During adolescence, the HPG axis undergoes complete reactivation.

The most important GnRH-inhibitory systems are gabaergic (neurons that produce gamma-aminobutyric *acid)* and opioidergic; Kisspeptin, neurokinin B and dynorphin A, three neuropeptides present in the arcuate nucleus (ARC), are considered to be fundamental generators that influence GnRH release, as they contribute significantly to the physiology of puberty in boys and girls (2).

The role of leptin in the physiology of puberty is well known. Leptin is a cytokine produced mainly by adipocytes, which acts as an anorectic factor, playing an essential role in controlling body weight, food intake, and energy balance by inhibiting the hypothalamic neuropeptide Y (NPY), thus suppressing appetite (13, 14). Normal body weight and composition must be attained during childhood to avoid pubertal dysfunction (1). In addition to the leptin-NPY interaction, some studies showed that leptin acts on puberty and reproductive function by directly interacting with the KiSS-1gene. GnRH neurons lack leptin receptors, but KiSS-1 neurons express them. Leptin directly stimulates kisspeptin release and mediates the pulsatile release of GnRH (15).

Physical changes occurring in puberty in male and female adolescents and the assessment of secondary sexual characteristics (breast buds in girls, testicular volume in boys, pubic hair in both), were classified according to the Marshall and Tanner classification

### Precocious puberty

Puberty is a complex process with wide physiological variation. Mechanisms regulating the onset of puberty involve genetic, nutritional and environmental interactions (1).

Abnormal fetal nutrition along with the endocrine system could lead to developmental alterations that permanently affect structure, physiology and metabolism. Interactions between hormones and nutrition during crucial periods of growth are essential concerning metabolic adaptation response control and pubertal development expectation (16).

An increasing amount of evidence suggests that the prenatal and early postnatal periods represent an important period for the programming of puberty onset (17). Various studies have shown that prenatal exposure to unfavorable environmental factors, such as factors responsible for children born SGA (small for gestational age) and/or IUGR (intrauterine growth restriction), have an effect on puberty timing (18, 19). A child born SGA may undergo several puberty alterations, such as precocious puberty (20–22).

Precocious puberty (PP) is defined by the early appearance of secondary sexual characteristics before the age of 8 years in female adolescents and 9 years in males (23). According to the underlying physiopathological process, pathological PP is classified as follows:

• Central precocious puberty (CPP) or gonadotropin-dependent PP (or true precocious puberty) caused by an early maturation of the HPG axis due to congenital or acquired central nervous system (CNS) lesions or monogenic defects, or it can be idiopathic (1);
• Peripheral precocious puberty (PPP) or gonadotropin-independent PP (or pseudoprecocious puberty), due to an excessive secretion of gonadal sex hormones or adrenal hormones from a genetic or tumoral etiology, germ cell tumors secreting hCG (human chorionic gonadotropin—exclusively in boys), or an exogenous source (1).

It has been evaluated that CPP affects 1 in 5000-10000 children, and is 10 times more common in females than in males (24). In addition, most of the cases of CPP in females seem to be idiopathic (25), whereas a higher prevalence of CPP in males seems to be commonly caused by pathological brain lesions (26). In particular, hypothalamic hamartoma is the most common brain lesion causing CPP.

There are some reports of familial forms, but the genetic basis is not completely understood. Some studies have shown associations with mutations (loss or gain of function) of KISS1 and makorin ring finger (MRF3) genes and their receptors. Mutations in these genes result in CPP; on the contrary, in familial CPP, *MKRN3* defects were found in approximately 30% of families in subjects with apparently sporadic CPP, and *MKRN3* defects were noted in approximately 8% of cases (27).

Patients with PP show accelerated sexual and physical growth concurrently with a growth spurt. If untreated, the accelerated epiphysial growth could lead to a short stature in adulthood due to premature epiphysial closure.

An accurate personal and familiar history, a complete physical examination, hormonal, and radiological exams is crucial in the PP diagnosis (23, 24, 28, 29).

The clinical examination should be focused on the auxological data, the assessment of pubertal signs according to the Marshall and Tanner classification (12, 30), the growth pattern during the last 6–12 months, the rate of progression of pubertal signs and additional signs of puberty (acne, oily skin, erections, nocturnal emissions in boys and vaginal discharge and menstrual bleedings in girls).

The baseline LH level is a promising biomarker to diagnose CPP (31); a basal morning LH value of more than 0.2 mUI/ml is usually considered indicative of puberty (28, 31–37). In addiction, an LH to FSH ratio higher than 0.6 has been associated with CPP (31, 34). Moreover, the GnRH stimulation test remains the gold standard to identify CPP, and the cutoff peak LH level of >5 IU/L is widely used to diagnose CPP (come sopra).

Other hormonal evaluations should include thyroid tests, testosterone, estradiol, 17-hydroxyprogesterone (17-OHP), carcinoembryonic antigen (CEA), Cancer antigen 125 (CA125), alpha-fetoprotein and beta-hCG depending on the patient’s history (38).

To define the biological age of the child, a bone age X-ray of the nondominant (left) hand and wrist is taken. An advanced bone age of more than 2.5 standard deviations (SD) or more than 2 years is more likely associated with pathological PP (28, 35).

In girls, pelvic ultrasound is a useful tool to assess the premature pubertal development of ovaries and exclude the presence of ovarian cysts or tumors (39, 40).

Brain MRI is suggested in patients with a CPP diagnosis to rule out CNS lesions (28), that should be performed routinely in young boys (< 6 years) (28).

The most important goals of the PP treatment are to preserve the adult height and to reduce the associated psychosocial stress.

GnRH agonists, with 1-month or 3-month depot formulations, are the standard of care in CPP. GnRH agonist therapy is widely considered safe. The most common adverse reactions include local skin effects and postmenopausal symptoms (35). The periodic verification of pubertal progression, growth velocity, and skeletal maturation is required.

The treatment of PPP varies according to the pathogenesis, and the primary aim of treatment is to eliminate the endogenous or exogenous sources of sex steroids (41, 42). Surgery is indicated in adrenal and gonadal tumors.

## Gut–sex hormones axis

The human intestinal tract is colonized by a large number of microorganisms (circa 10^13^-10^14^), known together as “microbiota”. The gut microbiome is the human body’s major ecosystem, so much so that according to some authors, it represents a “separate organ” (43). The human colon can be populated by more than 1000 different bacterial species and, each host can boast at least 160, which, however, vary in type and quantity depending on the person’s own health (44–46). This element underscores the interdependence that exists between host and GM, which affects multiple aspects of host health, particularly endocrine, gastroenterological (digestive function and intestinal permeability), and immune (resistance to foreign pathogens and stimulation of immunity) (47). Microbiota interact with a variety of metabolic and endocrine pathways of the host through genetic expression of more than 100 times the human genome. GM’s variety, composition and impact on health depend on a great number of variables, both internal, such as age, genetic factors, gender, and endocrine and immune systems, as well as external factors, such as diet, environment, drugs, and pathogens. All together they influence the delicate balance of the intestinal microecological system. In addition, research has shown that an imbalance in the GM can lead to a range of related diseases, especially those of autoimmune origin (5, 47).

In healthy subjects, more than 90% of bacteria are part of *Firmicutes* or *Bacteroides*’ phyla, and alterations in microbiota diversity are related to adverse outcomes in the host’s health. A decreased *Firmicutes/Bacteroides* ratio correlates with health issues such as obesity and immunological diseases (4, 48). Some authors have reported an association between an incremented *Firmicutes/Bacteroides* ratio and type 1 diabetes mediated by cell junction disruption with incremental gut permeability, bacterial translocation and the subsequent expression of pro-inflammatory cytokines (49).

The relationship between sex hormones and GM has been widely explored in recent studies and is an expanding research field that may lead to new therapeutic options for a great variety of sex-related diseases; the cluster of the gut microbiome’s genes capable of influencing sex hormone levels has been named the “microgenderome”.

One of the earliest studies was performed at the cellular level in the 1980s, in which it was found that progesterone promoted the growth of *Bacteroides* species and *Prevotella intermedia* (50, 51). Recently, Yurkovetskiy et al. (52) sequenced bacterial DNA extracted from the cecal contents of prepubertal (4 weeks old) and postpubertal (10-13 weeks old) mice and found that α-diversity was not significantly different between the sexes in prepubertal mice, which was evident among postpubertal mice. Furthermore, by sequencing the 16S rRNA genes of the microbiota of males, females, and castrated males, they observed that the microbiome of females was closer to that of castrated males than that of uncastrated males (50, 52). Among the most recent studies evaluating the relationship between microbiota and sex hormones, Elin Org et al. (53) further demonstrated the effect of androgens on microbiota composition, particularly by assessing the effects arising from gonadectomy and hormone supplementation (53). In contrast, there are still few studies conducted in humans evaluating the relationship between estrogen fluctuation and gut microbiome composition (4, 48). Moreover, these studies have an important limitation dictated by interfering factors, such as genetics and the environment, so most results can only support the existence of a correlation between sex hormones and the microbiome, rather than a causal relationship (50). Koren et al. (54), when sequencing stool samples from 91 women, observed that the gut microbiome was markedly altered during pregnancy, especially during the third trimester, when estrogen peaks, regardless of health status (50, 54). A European study by Mueller et al. (55) showed that healthy males had a higher abundance of *Bacteroides-Prevotella* than fertile females, while the microbiota of postmenopausal women did not differ from that of males (50, 56). Both studies demonstrate how estrogen and related female hormones are crucial in regulating the composition of the gut microbiome.

Therefore, it is well known that microbiota can affect estrogen levels and that, in turn, estrogen levels may be influenced by microbiota composition and diversity. Microbiome is capable of metabolizing estrogens *via* the expression of B-glucuronidase, an enzyme that mediates the deconjugation of dietary and non-dietary estrogen. Unconjugated estrogen can enter the systemic flow and become metabolically active by acting on alpha and beta estrogen receptors, which are expressed in a variety of organs and tissues; estrogen activity has an impact not only on reproductive health but also on cardiovascular risk, metabolic and bone homeostasis and the central nervous system (49, 50). Microbiota diversity is associated with higher urinary estrogen levels in postmenopausal women and in men, whereas in premenopausal women, estrogen levels do not seem to be influenced by microbiota composition, suggesting that microbiota mostly influences the levels of non-ovarian estrogens. The supplementation of phytoestrogen is capable of promoting gut colonization from specific bacterial species, and a phytoestrogen-rich diet may be associated with a lower risk of metabolic syndrome in Asian postmenopausal women (57, 58).

As already stated, the microbiome also influences the level of androgens in the host’s organism, and this might occur through a similar mechanism to that observed in women; a recent study found the levels of non-glucuronidated dihydrotestosterone to be lower in the distal intestine of germ-free mice compared to mice with normal microbiota, thus suggesting that intestinal bacteria express genes capable of metabolizing human sex hormones (59).

In turn, the sex hormone level may also affect microbiome composition: androgen excess, as in PCOS patients, may also lead to dysbiosis and lower bacterial diversity. PCOS is an endocrine disease characterized by higher androgen and lower estrogen levels, and several studies associated intestinal dysbiosis in PCOS patients with lower bacterial diversity, resulting in reduced butyrate production, higher BMIs and higher testosterone serum concentrations. Additionally, the gut microbiome plays an important role in determining insulin secretion by producing SCFAs, which help to reduce the inflammatory response; dysbiosis may lead to insulin resistance and alterations in glucose metabolism, as in polycystic ovary syndrome; higher insulin levels stimulate the ovary in producing androgens, thus perpetuating the pathogenetic mechanism of PCOS (48).

Microbiota and their metabolites may also affect every stage of female fertility, pregnancy, embryo development and timing of delivery, by colonizing the vaginal tract and, according to some authors, the endometrium and placenta. Microbiota’s alterations have been associated with the secretion of proinflammatory cytokines and preterm delivery (60). Neonates born from cesarean section delivery show lower diversity in intestinal bacteria, probably because they have not been colonized by maternal intestinal flora by passage through the vaginal tract (48).

Another possible mechanism for explaining microbiota’s influence on sex hormone levels involves the “gut-brain axis”, the two-way communication pathway between gut and CNS, according to which gut bacteria are an important mediator between the brain and the endocrine system (4). GM is central in modulating the brain–gut axis, and the gut barrier SCFAs, such as acetate, propionate, and butyrate, besides being modulators of inflammation capable of regulating gut motility and wound healing, represent a link between the microbiome and the gut–brain axis (4, 5, 61–63). In addition, the incidence of functional gastrointestinal disorders, such as functional dyspepsia and irritable bowel syndrome, resulting in impaired motility and/or altered sensitivity, are significantly higher in females, presumably due to a complex interaction between sex hormone signaling and stress reactivity in brain–gut axis function (6, 64). Specifically, estrogens have been observed to interfere with gastrointestinal motility and sensitivity through the direct activation of their receptors, located throughout the brain–gut axis, and indirectly through the modulation of other receptor systems (6). Gastrointestinal motility is reduced in women during the follicular phase of the ovarian cycle, when estrogen levels are high (6, 65). Furthermore, supporting the hypothesis that circulating female hormones play an important role in delayed gastric emptying, hormone replacement therapy administered to pre- and postmenopausal women correlates with a slower rate of gastric emptying than that of postmenopausal women not receiving hormone therapy, which in turn is similar to that of men of the same age (66). In contrast, testosterone, or androgens in general, appear to have no effect on gastric motility or gastric hypersensitivity (67, 68).

Estrogens implement their long-term mechanism of action through actions on nuclear receptors and rapid, nongenomic action through the activation of estrogen receptor 1 (ER 1) receptors coupled to membrane G proteins (6, 6). ERs are ubiquitously expressed in the CNS and in pathways involved in visceral pain perception, including the hypothalamus, amygdala, and midbrain, all of which have been shown to send extensive projections to vagal neurons involved in the modulation of gastrointestinal function (6, 69–71). Specifically, in peripheral visceral afferents, estrogen appears to modulate nociception by altering the opening of ion channels and the regulation of receptor expression, as well as activating the cholic tachykinin neurokinin 1 receptor and inducing the release of substance P (6, 72, 73). In addition to interfering with pain modulation, finally, estrogen is involved in visceral information processing in the CNS. Cerebral imaging studies have found that, in comparison with males with IBS, females with IBS display a greater activation of emotional circuits, including the amygdala and locus coeruleus, in response to adverse visceral stimuli (74).

Furthermore, several studies have demonstrated better cognitive functions and reductions in psychiatric symptoms in selected patients treated with fecal transplant. More studies on humans are needed to better understand the underlying mechanisms of this axis (4, 75).

**Figure 1:**
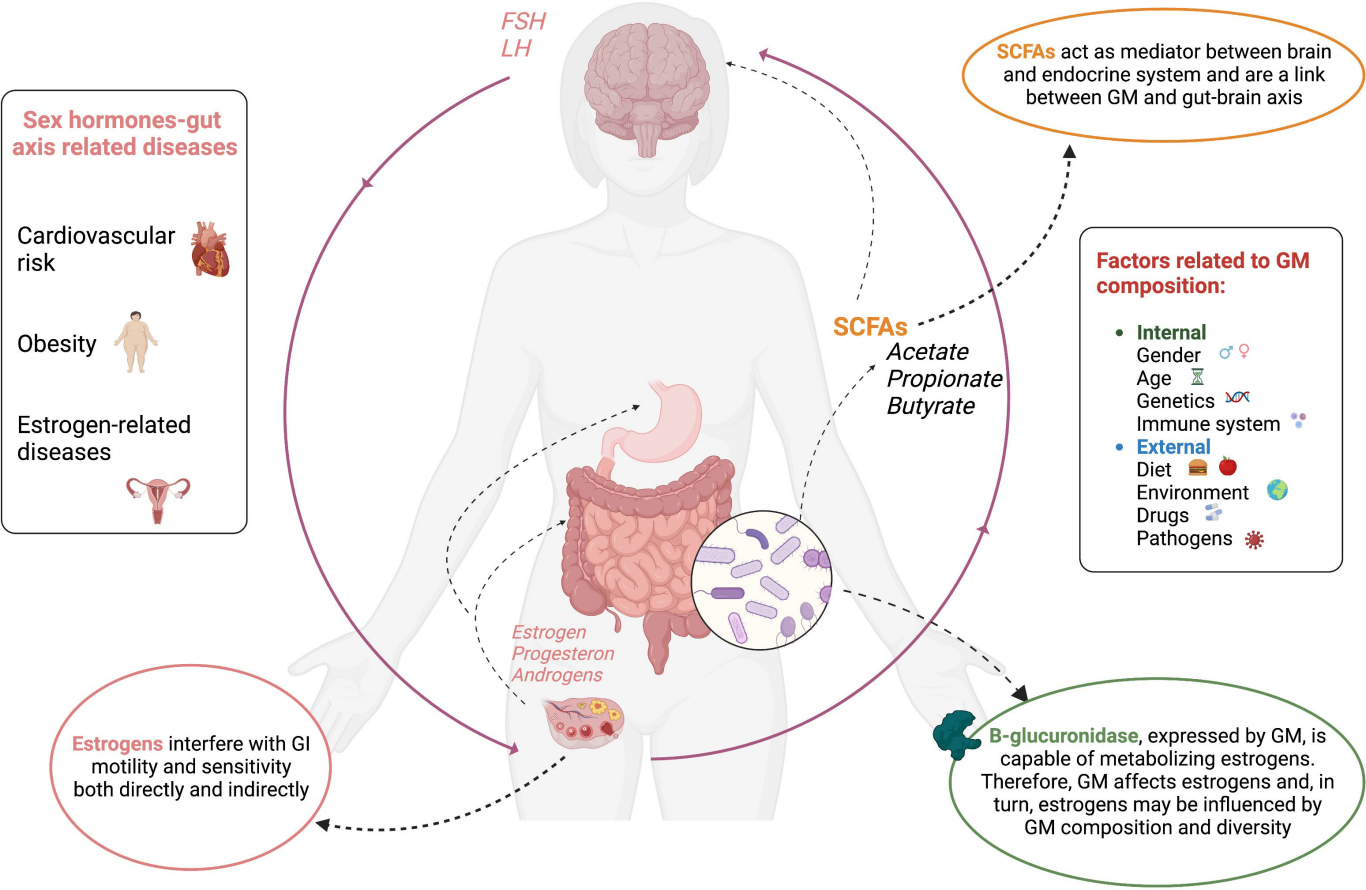
Interconnections between sex hormone and gut microbiome.

In **Figure 1**, the sex hormone–gut microbiome axis is shown.

## Microbiome in physiological pubertal development

It is well known how a gradual change in microbiota composition occurs with age with a general reduction in the number of aerobes and facultative anaerobes and an increase in the populations of obligate anaerobic species. Traditionally, the common idea is that between one and two years of age, the human GM starts to resemble that of an adult, which is dominated by species from phyla *Firmicutes*, *Bacteroidetes*, *Proteobacteria*, and *Actinobacteria.* However, many differences were observed at the genus level between adolescent and adult fecal microbiota (3, 7).

Agans et al. (7) conducted a study to assess the distal GM of adolescent children, which showed that abundance of members of *Bifidobacterium* and *Clostridium* genera, species known to colonize the newborn gut and to decrease gradually between 2 and 18 years of life, was statistically significantly higher in adolescent children than adults. The prevalence of these genera had not been recognized previously among preadolescent and adolescent age groups (7).

In 2020, Yuan et al. (76)conducted a cross-sectional study analyzing the biodiversity of the GM at different puberty stages (5-15 years) through 16S rRNA sequencing. No difference in alpha or beta diversity between non-pubertal and pubertal subjects was found, but the study evidenced differential bacterial taxa between the two groups. In particular, non-pubertal subjects were characterized by mainly microorganisms belonging to the order *Clostridiales*, family *Costridiaceae*, and genus *Coprobacillus*. On the other hand, the puberal group showed a higher prevalence of class *Betaproteobacteria*, order *Burkhollderiales* (76). Further analyses of the association between serum sex hormones and bacterial abundance were conducted. The results highlight that the level of testosterone was associated with the abundance of *Adlercreutzia, Dorea, Clostridium* and *Parabacteroides* genera. Authors hypothesized that these bacteria might be affected by sex hormones (76). Several studies have investigated the connection between gut microbes and sex steroid hormones. Shin et al. (77) demonstrated a relationship between intestinal bacterial community profiles and testosterone/estrogen status in humans: *Acinetobacter, Dorea, Ruminococcus* and *Megamonas* were significantly correlated with testosterone levels, while *Slackia* and *Butyricimonas* correlated significantly with estradiol levels (77).

It has been reported that GM does not seem to be affected by gender in children, but differences emerge at puberty onset (59). Microbiota-related diseases also show a gender bias, supporting that the relationship between intestinal bacteria and gender may be biunivocal (4). Although data on adolescents’ GM are still limited, a recent cross-sectional survey found that the distinction of the GM between the two sexes becomes more marked at puberty (78, 79). Comparing teens’ and adults’ microbiota, it was found that the amounts of bifidobacteria, in particular, decreased with age in several studies (7, 80–82), and age-related associations with *Bacteroidetes* and *Firmicutes* (80–82)were also reported (79). Hollister et al. (81) compared puberal and adult GM composition, pointing out that, during pubertal development, GM in girls changes progressively, increasingly resembling that of adult women, directly proportional to their pubertal developmental degree. In both males and females, dominant taxa are *Clostridia* and *Bacteroidia*, and the major element suggesting maturation of microbiota is represented by a change in the dominance of the *Clostridiales* and *Bacteroidales* classes of bacteria. Indeed, during puberty, the relative abundance of *Clostridiales* gradually increases and that of *Bacteroidales* decreases, gradually resembling the composition of the adult microbiota. In terms of phylum, the abundance of *Firmicutes* increased as puberty progressed, while the abundance of *Bacteroidetes* decreased. However, although Hollister et al. (81) observed GM changes in both boys and girls, only in female adolescents statistically significant data were obtained, reasonably due to later males’ pubertal development (81).

These data were confirmed by Yuan et al. (78) who determined the characteristics of the GM of both genders at different pubertal status. The GM was analyzed in 89 Chinese participants aged 5–15 years. Participants were divided into prepuberty and puberty groups for both males (n=49) and females (n=40). This cross-sectional study revealed that sex differences in the GM composition and predicted metabolic profiles existed before puberty and it became more significant in puberty. Specifically, results indicate that *Dorea, Megamonas, Bilophila, Parabacteroides* and *Phascolarctobacterium* genera represent microbial markers for pubertal subjects (78). They suggested that sex-dependent GM diversity is, in part, due to sex hormones, and, in part, to other non-hormonal influencing factors (78).

## Gut microbioma and precocious puberty

Considering evidences on the role of the GM during physiological pubertal development, their role in pathological puberty is becoming of increasing interest. As reported (83), timing of puberty can be influenced by GM, particularly by certain *Clostridia* species, including species of the genera *Ruminococcaceae Faecalibacterium* and *Ruminococcus*, which regulate host sex hormone levels. Specifically, these species affect estrogen metabolism through their beta-glucuronidase activity (81). The beta-glucuronidase enzymes of *Ruminococcus* and *Faecalibacterium* spp. are able to cleave both estrone and estradiol, whereas *Bacteroides* species are only capable of metabolizing estrone. Therefore, estrone-estrogen metabolite ratio in urine correlates positively with the relative abundance of *Ruminococcus* and negatively with that of *Bacteroides* spp (81, 84). According to these data, it is conceivable that the GM may partly regulate the onset of puberty through its estrogen metabolism. However, as much as the GM, through specific gut microbes capable of metabolizing estrogen, seems capable of regulating puberty, the reverse may also be possible. In fact, sex hormones could directly affect the growth of specific taxa by directing the maturation of the gut microbiota (81).

Furthermore, recent studies have observed that metabolites produced by the GM could influence the human endocrine system, activating the enteric nervous system. Some of the best studies on microbiota functions highlight how gut microorganisms provide energy to the host through the production of short-chain fatty acids (SCFAs), including butyrate (the most abundant SCFA) and propionate, both of which participate in bile salts metabolism and play an important role in brain-gut axis (4, 48). It has been demonstrated that Free Fatty Acid Receptor (FFAR) 2 and FFAR3, endogenous receptors, interact with the SCFAs and have been shown to be expressed in enteroendocrine cells that produce peptide YY, an anorectic hormone, with consequent involvement in the regulation of the host energy, appetite, adipose tissue stores and hormonal balance, influencing puberty timing (85, 86)

Indeed a close association between obesity and puberty has been found; in particular, PP has been positively related with body mass index (BMI). On the basis of this information and given that children affected by PP tend to be obese, it has been hypothesized that GM could be involved in the pathogenesis of PP. The study conducted by Dong et al. (87) elucidated differences in the GM between patients with idiopathic central precocious puberty (ICPP) (n=25) and healthy girls (n=23). Authors applied 16S rDNA sequencing to compare the GM between two groups. They observed that the gut genera identified in ICPP are similar to those that are associated with obesity, in particular, *Rumicoccus Gemmiger, Oscillibacter* and *Clostridium XIVb*. Considering microbial species levels, girls with ICPP were enriched in *Rumicoccus bromii, Ruminococcus gnavus*, and *Ruminococcus leptum.* The first two were found in obese populations; they could promote the energy absorption and hyperplasia of adipose tissue, while *Ruminococcus leptum* was reported to influence human weight changes (87–89). These results highlight the association among obesity, ICPP and GM dysbiosis. The authors hypothesized that GM dysbiosis leads preadolescent girls to a process similar to that in obese patients and that the proliferation and deposition of adipocytes trigger precocious puberty. However, intestinal dysbiosis could also induce the earlier activation of the hypothalamic–pituitary–gonadal axis (HPGA) (87).

The involvement of the GM in the mechanism of secretion of estrogen, FSH and LH has been investigated in different studies, but it is still unclear. A previous study indicated a relationship between estrogen and bacteria, such as *Clostridia* and *Ruminococcaceae*, and a significant microbial differences among the control group and ICPP (90). Dong et al. (87) explored the relationship between three clinical biomarkers (FSH, LH and insulin resistance) and the GM. Considering ICPP girls, authors demonstrated a positive correlation between FSH and *Fusobacterium*, and LH and *Gemmiger*, and a negative correlation between LH and *Romboutsia* (87). In addition, insulin resistance has been positively correlated with *Gemmiger, Ruminococcus, Megamonas* and *Bifidobacterium* (87).

In female puberty onset, the important role of leptin is well known. Leptin is an adipocyte metabolic peptide, and the gene involved in its expression is correlated with SCFAs (83, 85, 88, 91, 92).

The close association among the GM, hormone secretion and obesity inspired the study of the mechanism of the GM in triggering CPP. ICPP girls investigated in the work by Dong et al. were characterized by microbes associated with SCFA production: *Ruminococcus bromii, Ruminococcus callidus, Roseburia inulinivorans, Coprococcus eutactus, Clostridium sporosphaeroides* and *Clostridium lactatifermentans*. The relationship between SCFA production and ICPP is explained through the mechanism induced by a high concentration of SCFAs to the expression of the leptin gene, which activates the HPGA, which, consequently, leads to the onset of puberty (87).

**Figure 2:**
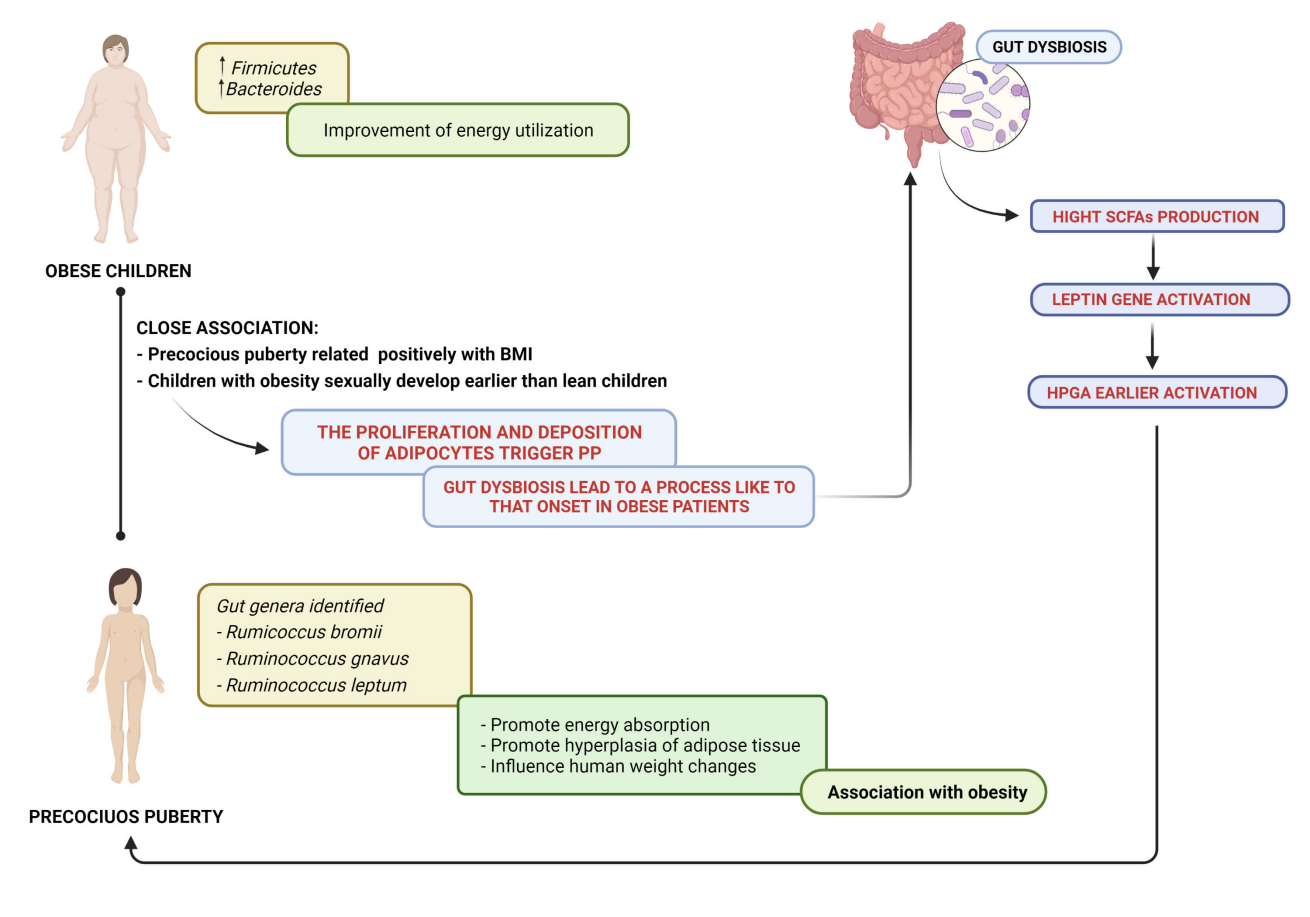
Precocious puberty–gut microbiome and obesity: a close association. SCFAs, short-chain fatty acids; HPGA, hypothalamic–pituitary–gonadal axis.

Li et al. (93) enrolled 27 CPP girls, 24 overweight girls and 22 healthy controls to explore the connection between obesity and CPP. This study showed that CPP patients exhibited overrepresented *Alistipes, Klebsiella* and *Sutterella*, which are normally present in patients with neurological diseases. These microorganisms produce metabolites with neurotransmission activity (serotonin and dopamine), which trigger the earlier onset of puberty activating HPGA. The authors identified *Prevotella* both in CPP and in the overweight group; branched-chain amino acid production could promote insulin resistance. This mechanism could explain the high occurrence of obesity in CPP patients (93). In addition, in both groups, elevated nitric oxide synthesis was observed, which is an important gas neurotransmitter that stimulates the secretion of gonadotropin-releasing hormone and promote insulin resistance (93). These conditions, the altered expression of the GM, could explain the link between CPP and obesity (93), as shown in **Figure 2**.

As reported (1), the macronutrient food content, such as high fat intake, may modulate the premature activation of the HPG axis, inducing precocious activation of puberty. Recently, in experimental model Bo et al. (94) showed that the effect of high-fat diet (HFD) on precocious puberty is regulated by the interaction of gut microbiota and hormones. HFD after weaning caused PP, increased serum estradiol, leptin, deoxycholic acid and GnRH in the hypothalamus (94). In particular, GnRH was positively correlated with *Desulfovibrio, Lachnoclostridium, GCA-900066575, Streptococcus, Anaerotruncus*, and *Bifidobacterium*, suggesting that these bacteria may have a role in promoting sexual development (94). Additionally, the authors (94) reported that “HFD-microbiota” transplantation promoted the PP of mice, supporting that GM modulates local and systemic levels of sex steroids promoting precocious puberty.

## Conclusions

Bidirectional interactions between the GM and the sex hormones have been proposed in different studies. During puberty, the somatic developmental changes are predominantly driven by hormones; therefore, this dynamic and transitional period represents an opportunity to assess the impact of potential hormonal effects on the GM. Although the evidence of the interaction between microbiota and sex hormones remains limited in pediatric patients, the evidence that diversity of the GM at different puberty stages exists and that GM alterations may occur in girls with CPP represents an interesting finding for the prediction and prevention of precocious pubertal development. Deepening the understanding of the connection between the sex hormones and the role of microbiota changes can lead to the implementation of microbiota-targeted therapies in pubertal disorders by offering a pediatric endocrinology perspective.

## Author contributions

VC, VR, GM, CR, CH, SP, CB, GZ participated in the study design, project management, and supervision. VC, VR, GM, CR, CH were responsible for the conceptualization and design of forms, data management, writing, and editing. VC, SP, CB, GZ supervised the manuscript. All authors were contributed to this article and approved the submitted version.

## Conflict of interest

The authors declare that the research was conducted in the absence of any commercial or financial relationships that could be construed as a potential conflict of interest.

## Publisher’s note

All claims expressed in this article are solely those of the authors and do not necessarily represent those of their affiliated organizations, or those of the publisher, the editors and the reviewers. Any product that may be evaluated in this article, or claim that may be made by its manufacturer, is not guaranteed or endorsed by the publisher.
